# Enhanced recovery after surgery review and urology applications in 2020

**DOI:** 10.1002/bco2.9

**Published:** 2020-03-17

**Authors:** Rodrigo Rodrigues Pessoa, Ahmet Urkmez, Naveen Kukreja, Janet Baack Kukreja

**Affiliations:** ^1^ Division of Urology University of Colorado Aurora CO USA; ^2^ Department of Urology University of Texas MD Anderson Cancer Center Houston TX USA; ^3^ Department of Anesthesia University of Colorado Aurora CO USA

**Keywords:** enhanced recovery after surgery, fast track, perioperative care, surgical recovery

## Abstract

**Purpose:** To explore enhanced recovery after surgery (ERAS) components and their current application to major urologic surgeries, barriers to implementation and maintenance of the associated quality improvement. **Data Identification:** An English language literature search was done using PubMed. **Study Selection:** After independent review, 55 of the original 214 articles were selected to specifically address the stated purpose. **Data Extraction:** Clinical trials were included, randomized trials were prioritized, but robust observational studies were also included. **Results of Data Synthesis:** Many ERAS components have good data to support usage in radical cystectomy (RC) patients. Most ERAS programs include multidisciplinary teams carrying out multimodal pathways to hasten recovery after a major operation. ERAS components generally include preoperative counseling and medical optimization, venous thromboembolism prophylaxis, ileus prevention, avoidance of fluid overload, normothermia maintenance, early mobilization, pain control and early feeding, all leading to early discharge without increased complications or readmissions. Although there may not be specific data pertaining to other major urologic operations, the principles remain similar and ERAS is easily applicable. **Conclusion:** The benefits of ERAS programs are well established for RC and principles are easily applicable to other major urology operations. Barriers to implantation and maintenance of ERAS must be recognized to continue to maintain the benefits of these programs.

## 1 INTRODUCTION

Enhanced recovery after surgery (ERAS) has become an international urology movement toward perioperative programs to improve postoperative outcomes including accelerated recovery and decreased length of stay (LOS). This has been accomplished without an increase in readmission or complication rates. ERAS protocols aim at reducing postoperative stress, maintaining perioperative physiological functions and enabling early mobilization. This results in reduced morbidity, decreased recovery time, and shorter hospital LOS.^1^ Urology patients undergoing major surgery, specifically those undergoing radical cystectomy (RC), benefit from the applications of ERAS leading to improved perioperative care.^1^, ^2^ However, the application of ERAS principles are applicable to most major urologic surgeries. Important ERAS components include preoperative counseling, medical optimization, avoidance of fasting, avoidance of bowel preparation, venous thromboembolism (VTE) prophylaxis, avoidance of salt and water overload, maintenance of normothermia, appropriate antibiotic use, ileus prevention, postoperative nausea and vomiting (PONV) prevention, pain control, early mobilization and early oral nutrition with avoidance of nasogastric tubes (NG). In this review, we aim to present ERAS applications in major urologic surgeries, a detailed narrative of each component and useful guidance for implementation.

## 2 METHODOLOGY AND LITERATURE SEARCH PRIORITIZATION

A review of the literature was performed to create this narrative review using the PubMed database through November 2019 to identify original and review articles regarding ERAS protocols in abdominal and urologic surgery. Key words used in the search are listed in Supporting Information 1. Supporting Information 2 contains the flowchart of study selection.

**Figure 1:**
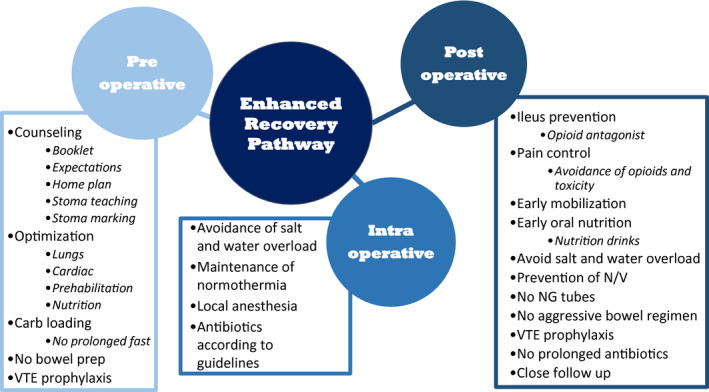
This is a schematic diagram of enhanced recovery. Important components are divided into preoperative, intraoperative and postoperative categories. VTE, venous thromboembolism; NG, nasogastric; N/V, nausea and vomiting. From Baack Kukreja et al BJUI 2017^5^

ERAS is often divided into three phases: preoperative, intraoperative and postoperative. All contacts who influence the patients' care throughout the health system are generally included in development and implementation in their respective segment. A schematic diagram of ERAS is seen in Figure 1.^2^ After primary review and initial screening of 214 abstracts, 120 articles with full texts were identified. After the secondary review and assessment of full texts, 55 articles regarding major urological and abdominal surgeries were selected. Two additional manuscripts were added after reference list review. Therefore, a total of 55 articles were included to specifically address the stated purpose. Table 1 lists an overview of the major studies included in this review and the key findings.

**Table 1:** ERAS in urology

ERAS elements	Studies	Year	Methods	Patients	Primary outcome	Complication
ERAS protocol	Daneshmand et al.^1^	2014	Prospective	110 w ERAS (75 w continent UD, 35 w non‐continent UD)	Median LOS 4 days, 82% had bowel movement PO 2. Day	The 30‐day readmission rate and major complication rates were 21% and 14%, respectively
	ORC				>75 years patients had longer LOS (5 days)	
ERAS protocol	Kukreja et al.^2^	2017	Prospective	79 w ERAS, 121 w/o	LOS 5 vs 8 days (*P* < .001)	No increase in readmission or complication rates
	ORC and iRARC					
Prehabilitation	Minella et al.^8^	2019	RCT	35 w prehabilitation, 35 standard	4 weeks after surgery, functional capacity (6MWD) 142.5 vs 123.8 m (*P* = .014)	
	ORC					
Mechanical Bowel Preparation (MBP)	Raynor et al.^9^	2013	Prospective	33 w/o MBP, 37 w MBP	No difference in GIS complications (15% vs 22%, *P* = .494)	No occurrences of anastomotic leak, fistula, abscess, peritonitis or surgical site infection
	ORC					
Alvimopan	Sultan et al.^13^	2017	Sys‐ Rev	143 w Alvimopan. 137 w placebo	Reduced time to bowel movements (HR 1.77, 95% CI, 1.41‐2.23). Reduced LOS (HR 1.67, 95% CI 1.38 to 2.01)	Reduced major adverse events (RR 0.28, 95% CI, 0.18 to 0.44). No increase in readmission rates
	ORC		1 RCT			
Extended VTE prophylaxis (ETP)	Naik et al.^17^	2019	Sys‐ Rev	23 with TR	VTE risk is highest in ORC and RARC (2.6‐11.6%). For ORC and RARC, ETP significantly reduces VTE risk but not PE risk. No data related to VTE risk reduction w ETP in RP and nephrectomy	Does not significantly increase bleeding risk for most major urological operations. Individualized risk assessment should be done
	ORC		3 prospective	24 w/o		
	RARC		9 retrospective			
	ORP					
	RARP					
	OPN					
	ORN					
	RARN					
	RAPN					
Non opioid protocol	Audenet et al.^23^	2019	Prospective	52 w non‐opioid	Reduced PO morphine (2.5 vs 44 mg, *P* < .001). Reduced LOS (5 vs 7 days, *P* < .001) Reduced median time to regular diet (4 vs 5 days, *P* = .002)	No difference in pain scores and complication
	eRARC			41 w opioid		
Surgical technique	Tan et al.^27^	2018	Prospective	45 ORC w/o ERAS	Shorter LOS in iRARC w ERAS compared iRARC w/o ERAS (7 vs 11 days). The median LOS in ORC w/o ERAS was 17 days	Significantly lower 90 days readmission rates (*P* < .001) and GIS complications (*P* = .001) in iRARC w ERAS group
	ORC			50 iRARC w/o ERAS		
	iRARC			50 iRARC w ERAS		
Pelvic drainage	Kowalewski et al.^35^	2019	Sys‐Rev	3664 patients	For RP, a reduction in PO complications w/o drainage (OR 0.62, CI 0.44;0.87, *P* = .006)	For RP and PN, no differences in readmission, re‐intervention, lymphocele, hematoma, urinary retention and overall complications. For RC, little evidence for recommendation
	ORP		6 study for RP			
	LARP		4 study for PN			
	RARP		1 study for RC			
	RAPN					
	OPN					
	LPN					
	ORC					

Abbreviations: 6MWD, 6 minute walking distance; eRARC, robot‐assisted radical cystectomy and extracorporeal diversion; ERAS, enhanced recover after surgery; iRARC, robot‐assisted radical cystectomy and intracorporeal diversion; LARP, laparoscopy assisted radical prostatectomy; LOS, hospital length of stay; LPN, laparoscopy assisted partial nephrectomy; OPN, open partial nephrectomy; ORC, open radical cystectomy; ORN, open radical nephrectomy; ORP, Open radical prostatectomy; RAPN, robot assisted partial nephrectomy; RARC, robot assisted radical cystectomy; RARN, robot assisted radical nephrectomy; RARP, robot‐assisted radical prostatectomy; RCT, randomized controlled trial; Sys‐Rev, systematic review; UD, urinary diversion; VTE, venous thromboembolism.

## 3 PREOPERATIVE COMPONENTS

### 3.1 Patient counseling

Preoperative counseling of patients can help to target expectations from the surgical and anesthesia teams. It may reduce fear, anxiety, fatigue, and pain and can contribute to accelerated postoperative recovery and early discharge.^3^

### 3.2 Stoma education and marking

Urostomy care requires manual skills and emotional adaptation. Patients receiving a preoperative and postoperative detailed stoma education with a stoma specialist in an ERAS program have shorter LOS (6 vs 9 days) without any difference in readmission rate or early stoma complications.^4^

### 3.3 Preoperative optimization of medical condition

Optimization of cardiac, pulmonary and kidney disease, hypertension, diabetes, anemia, nutrition, cessation of excessive alcohol use and smoking all improve perioperative outcomes.^5^ The pulmonary effects of smoking can be improved by quitting 4 weeks before surgery.^6^ Longer smoking cessation may improve wound healing and lower cardiovascular risks. Intensive preoperative interventions aimed at complete alcohol cessation at least 4 weeks before surgery may reduce postoperative complications by improving cardiac functions, blood clotting, immune functions and response to surgical stress.^7^ Therefore, we believe there has to be a combined effort between surgeons, primary care physicians, and anesthesiologists in order to counsel and refer patients at risk to dedicated smoking cessation services. Smoking cessation interventions include patient education (pamphlets/brochures, e‐learning/web‐based education), tobacco quitlines, text‐messaging systems, phone applications, face‐to‐face behavioral support and pharmacotherapy (nicotine replacement therapy, bupropion, varenicline).

### 3.4 Prehabilitation and exercise

Prehabilitation programs optimize a patient's preoperative condition to improve postoperative outcomes by promoting physical and psychological health and can be applicable to all patients undergoing major urologic operations. Although patients have a relatively short time to achieve optimization prior to surgery, a recent RCT in patients undergoing RC demonstrated home‐based multimodal prehabilitation (exercise, nutrition program and psychosocial support) provided faster recovery after RC.^8^

### 3.5 Mechanical bowel preparation

Omitting both mechanical and antibiotic bowel preparation in RC with small bowel urinary diversions has demonstrated no difference in terms of gastrointestinal (GI) complications and wound infections.^9^ Based on colorectal literature, it is recommended that patients undergo mechanical and chemical bowel preparations for diversions using the colon.^10^

### 3.6 Preoperative fasting and carbohydrate loading

Numerous studies have demonstrated that cessation of solid food and liquid intake, starting at midnight is associated with a number of metabolic derangements, including insulin resistance.^3^ Shortening this period improves patients' comfort and reduces the surgical stress response.^11^ Current guidelines by the ERAS society allows the intake of clear fluids up to 2 hours and solid foods up to 8 hours prior to surgery.^3^

In the Cochrane Database, a review including 27 trials, demonstrated that preoperative carbohydrate loading was associated with a slightly decreased hospital LOS without affecting postoperative complication rates when compared with fasting and placebo.^12^

### 3.7 Preoperative opioid antagonist administration

*Alvimopan is a peripherally acting* μ‐opioid receptor antagonist. Administration of oral alvimopan in RC patients has been associated with quicker GI recovery, shorter hospital LOS, decreased postoperative ileus‐related morbidity and reduced cost with a similar safety profile when compared to placebo.^13^

### 3.8 Pre‐anesthetic medications

Long‐acting anxiolytic premedication should not be routinely administered to patients as it may delay early postoperative recovery, mobilization and may cause cognitive impairment, particularly in elderly patients.^14^

### 3.9 Venous thromboembolism prophylaxis

VTE is the most common cause of the death within 30 days of urological cancer surgery.^15^ Most VTEs occur after discharge with a median time to develop VTE of 20 days.^16^

VTE risk after major urologic operations decreases with extended prophylaxis (EP) (28 days) when compared to standard prophylaxis. EP does not significantly increase bleeding risk for most major urologic oncology operations.^17^ For major urologic surgery, an individualized risk assessment should be completed and EP may be recommended for those with increased VTE risk. Mechanical thromboprophylaxis with compression stockings and/or intermittent pneumatic compression devices should be applied to all major abdominal surgery patients until discharge.^5^

### 3.10 Antimicrobial prophylaxis

Patients benefit from prophylactic antibiotics, although the optimal regimen is unclear and likely depends on local antibiotic‐resistance profiles. Antimicrobial coverage should include aerobic and anaerobic bacteria for patients undergoing bowel resection. A second or third generation cephalosporin or, alternatively, use of an aminoglycoside in combination with metronidazole or clindamycin is recommended to be administered within 1 h prior to skin incision.^3^, ^10^ In prolonged surgeries and in surgeries with increased blood loss, repeated dosages every 3‐4 hours are beneficial, depending on the half‐life of the drug.^18^ Despite adherence to evidence based guidelines, readmissions for infection after RC remains significant. The duration of antibiotics is not clear for RC, but readmissions are not reduced by prolonged antibiotic administration.^19^

### 3.11 Skin preparation

Skin preparation prior to surgery using a chlorhexidine‐alcohol scrub is suggested in ERAS guidelines to prevent surgical site infections (SSIs). However, in a Cochrane review, including >10 000 patients, authors reported no benefit for preoperative showering or bathing with chlorhexidine over other antiseptics in order to reduce SSIs.^20^

## 4 INTRAOPERATIVE COMPONENTS

### 4.1 Anesthetic protocols

Collaboration with the anesthetic team is critical. The anesthesia team is responsible for influencing the outcome of surgery in multiple ways including: stress response to surgery, fluids and analgesia. ERAS anesthetic protocols include the use of thoracic (T9‐11) epidural anesthesia (usually only needed for open surgery), minimal use of opioids, use of fentanyl‐based short‐acting opioids such as remifentanil when opioids are needed, along with strategies for prevention of hypothermia, hypoxemia and hypovolemia.^21^ In a recent study, authors investigated the effect of including anesthesia ERAS components to an existing surgical ERAS program for RC. They found a decrease in intraoperative transfusions and PONV.^22^ Implementing non‐opioid pain medications with regional anesthesia can decrease LOS, time to regular diet and decrease narcotic use.^23^ In 2016, the European Association of Urology's (EAU) Robotic Urology Section (ERUS) reported a consensus statement regarding ERAS after robot‐assisted radical cystectomy (RARC). The committee reached 89% consensus that epidural analgesia can be routinely omitted during RARC with intracorporeal urinary diversion (iRARC), and 75% agreed on routine omission with an extracorporeal urinary diversion.^24^

### 4.2 Surgical approach

Surgical approach (open vs minimally invasive surgery [MIS]) may impact outcomes, complications and recovery rates. MIS requires smaller incisions, reduces analgesic requirements, reduces bowel handling, and decreases blood loss.^25^ MIS has shown benefit in patients undergoing prostatectomy, nephrectomy and partial nephrectomy. MIS is also associated with a decrease in inflammatory response when compared to open surgery.^3^ A meta‐analysis comparing RARC and ORC found that cancer control outcomes are similar between the two techniques, while blood loss is lower in RARC, operative time is longer, however, complication rates are similar for both surgical approaches.^26^ In another study, authors compared ORC, RARC with iRARC with and without the implementation of an ERAS program; LOS was significantly reduced in iRARC compared with ORC. In addition, the addition of an ERAS program to iRARC further decreased the LOS (median 7 days with ERAS vs 11 days without ERAS) without increasing 90‐day readmission rates.^27^

### 4.3 Perioperative fluid management

The aim of the fluid management is maintaining intravascular volume, cardiac output and tissue perfusion while avoiding overload. Maintaining a net even fluid balance significantly decreases blood loss, LOS and overall complication rates.^28^ Fluid management in patients undergoing major urologic surgery can be challenging, as urine output is often not measurable intraoperatively. Goal‐directed fluid therapy (GDFT) can be used as an alternative by using a transesophageal doppler to monitor and optimize stroke volume. In a RCT of patients undergoing RC the incidence of ileus and PONV at 24 and 48 hours was significantly reduced with GDFT.^29^ In addition, perioperative restrictive hydration employed in conjunction with a norepinephrine infusion in the context of ERAS program did not influence postoperative renal functions when compared with liberal hydration and did improve complication incidence.^30^

### 4.4 Nasogastric tube placement

Avoidance of a NG is recommended for ERAS patients. A Cochrane meta‐analysis indicated increased postoperative complications without occurrence of any advantage if prophylactic NG tube was placed after major abdominal surgery.^31^ In another meta‐analysis, including 780 patients who underwent RC, while there were no differences in respiratory complications, the time of GI functional recovery and LOS were shorter in patients without a NG tube after surgery.^32^ EAU ERUS working committee reached 85% consensus that the NG tube can be removed at extubation.^24^ Therefore, NG decompression may be reserved to cases of prolonged postoperative ileus.

### 4.5 Prevention of intraoperative hypothermia

Intraoperative normothermia is critical as hypothermic patients have higher wound infection incidence, more cardiac events and more bleeding. Maintaining normothermia intraoperatively is likely to reduce the infectious complications and shorten LOS.^33^ Therefore, maintaining intraoperative normothermia with external heaters (forced air warming blankets) are strongly recommended and intravenous fluids given should be warmed.

### 4.6 Urinary catheter

There is currently no study evaluating the optimal timing of removal of ureteric stents after RC or the transurethral catheter following RC with orthotopic neobladder. For other major urologic operations it is important to keep in mind that early removal of a transurethral urinary catheter following reduces the incidence of urinary tract infections and shortens the LOS.^34^

### 4.7 Pelvic drainage

Prophylactic abdominal or retroperitoneal drain placement after major urologic surgeries has been the traditional standard of care. A recent meta‐analysis on prophylactic drain placement in major uro‐oncologic surgeries, including 3664 patients; showed that, for prostatectomy, postoperative complications were fewer in patients without drainage while there were no differences in incidences of re‐intervention, lymphocele, hematoma or urinary retention. Findings concluded for prostatectomy and partial nephrectomy, the placement of a drain can be omitted unless there is a deviation from the standard care. However, for RC, the evidence was insufficient to suggest drain omission.^35^

## 5 POSTOPERATIVE COMPONENTS

### 5.1 Nausea and vomiting

PONV is experienced by 25%‐35% of surgical patients. This is the major cause of mobilization difficulty, delayed oral food intake, delayed discharge and patient dissatisfaction. PONV increases the risk of pulmonary aspiration. The etiology may be patient specific, anesthetic‐related and surgery‐related. Female patients, non‐smokers, and patients with a history of motion sickness are at high risk. The use of inhalation anesthetics, nitrous oxide and opioids significantly increases the risk as well.^3^

In recent years, the multimodal approach to PONV has gained popularity. Non‐pharmacological and pharmacological antiemetic methods are applied together in ERAS programs. Minimizing preoperative anxiety, pre‐hydration with oral carbohydrate containing fluids, shortening preoperative fasting time, preoperative dexamethasone, avoidance of inhalation anesthetics are some of the factors that reduce PONV. The use of propofol in induction and maintenance of anesthesia may be considered in patients deemed at higher risk of PONV vomiting when appropriate. In addition, the use of regional anesthesia techniques (such as epidural, transverse abdominal plan block) reduces postoperative opioid usage and PONV. Pain itself increases PONV, therefore the aim should be creating the optimal balance between opioid administration and pain relief. Non steroid anti‐inflammatory drugs (NSAID) are also recommended as an alternative to opiate use.^36^ The effects are increased by the combined use of two or more antiemetics (up to four). Moreover, the risk of PONV decreases by 30% with each additional administered antiemetics. Antiemetics such as dexamethasone, ondansetron and neurokinin‐1 receptor antagonists are also helpful in reducing PONV.^36^

### 5.2 Early mobilization

Postoperative prolonged immobilization is associated with an increased risk of pneumonia, insulin resistance and muscle breakdown. Encouraging early postoperative mobilization is also important for avoiding pain and ileus.^5^ A recent prospective study evaluated the association between daily ambulation measured by wearable activity monitors and LOS among patients undergoing major surgery (including RC). They found that higher step count (up to 1000 steps) on postoperative day 1 was associated with a lower probability of a prolonged LOS.^37^

### 5.3 Ileus prevention

Postoperative ileus prevention after abdominal surgery is an important step in accelerated healing protocols. Mid‐thoracic epidural analgesia (compared to intravenous opioid analgesia), avoidance of intraoperative and postoperative fluid overload have been shown to be highly effective for preventing postoperative ileus.^1^, ^2^, ^3^ Alvimopan or other peripheral opioid antagonists are often critical to ileus prevention.^1^, ^2^ In a meta‐analysis of 18 RCTs, the addition of chewing gum was a safe and effective method in preventing postoperative ileus after bowel surgery and was associated with shorter LOS.^38^ In a population based retrospective study, including nearly 3.5 million patients (89 000 patients who underwent RC), evaluated postoperative ileus after major oncologic surgeries in the ERAS era. The highest ileus rate was recorded after RC (predicted probability: 26%). MIS was found to be associated with lower risk of postoperative ileus.^39^

### 5.4 Postoperative analgesia

Ideal analgesia regimens after major surgery should relieve pain, assist in early mobilization, aid in the return of GI function, allow for oral nutrition and not increase complications (postoperative ileus, PONV, etc.). Multimodal opioid‐sparing analgesia with regional or local anesthesia is recommended to provide effective pain management while minimizing the side effects of opioids.^40^ In a double‐blinded RCT, patients undergoing RARP were assigned to receive either intravenous 1gram acetaminophen or placebo within 15 minutes after induction of anesthesia and repeat doses of acetaminophen or placebo was administered every 6hours for four doses. In patients who received acetaminophen, hospital LOS was significantly shorter (by 32%) when compared to placebo and there were no differences in pain scores or opioid use.^41^ As part of an ERAS multimodal analgesia plan, ultrasound transversus abdominal plane block provided lower usage of opioids, improvement in time to flatus, and a shorter hospital LOS compared to conventional post‐RC pain management without observing any differences in complications or readmission rates.^42^ Local wound infiltration with long acting liposomal bupivacaine is used in some institutions, however RCT data in major urologic operations is lacking.

### 5.5 Early feeding

Postoperative early feeding is one of the most important components for postoperative well‐being and early discharge. Traditionally, it has been thought that starting early enteral nutrition could increase GI complications, however, a Cochrane review of 13 RCTs revealed that earlier feeding might reduce the risk of postsurgical complications.^43^ Additionally, early enteral nutrition (within 24 hours) has positive effects on insulin resistance, muscle function, and wound healing without increased morbidity.^44^ The effect of specialized perioperative nutritional interventions (immune‐enhancing nutrition, amino acids, multivitamin and mineral supplement, etc.) in patients undergoing RC has been evaluated, but small patient numbers limit the quality of evidence, however, there are promising ongoing RCTs in this area.^45^

### 5.6 Discharge

ERAS protocols allow for decreased LOS and earlier discharge. Discharge parameters that usually need to be met include pain control with oral medications, tolerating full diet with at least 1‐L oral intake per 24 hours, adequate mobilization and return of GI function.^1^, ^3^

### 5.7 Follow‐up

The aim of ERAS protocols is to provide rapid recovery and return to the baseline preoperative status. Although readmissions remain high in this area, ongoing work into decreasing readmissions is broadly being studied.^46^

## 6 HEALTH SYSTEM CONSIDERATIONS AND EFFICACY OF ERAS PROGRAMS

### 6.1 Barriers of implementation of an ERAS program

Healthcare's increasing complexity and expenditures continue to challenge providers and administrators. Identifying modifiable perioperative processes such as ERAS has the potential to maximize efficiency in delivery of care and improve patient outcomes at the same time. Even with increasing evidence demonstrating better outcomes, there are still several barriers to full adoption and implementation. The first layer of difficulty comes from urologists with well‐established systems who are ultimately required to change practice patterns at their respective institutions. On a recent survey among urologic oncologists, 64% always classified themselves as ERAS adopters but half of them omitted two or more of the core principles and only 20% endorsed using all them.^47^ Reasons for low actual application of core principles vary and range from concerns regarding lack of enough evidence in the literature, the idea that ERAS do not work, to lack of institutional support.^47^

Ideally, comprehensive assessment of baseline outcome data should be obtained before application of any protocol. This will provide data to be compared to once the benefits of the employed ERAS protocol start to mature. Any barriers to implementation should be identified, so that it can be determined locally what may delay further execution of the protocol. A thorough systematic review on ERAS implementation showed that there are multiple topics common to most institutions, even though the list of facilitators and barriers may vary considerably from one site to another.^48^ In general, lack of clear guidance was found to deter adherence, so standardization with some degree of flexibility to adapt the protocol to local factors is of paramount importance. Early stakeholder involvement is essential, along with networks of open communication where all involved participants of the multidisciplinary team can freely give their inputs, take ownership of the project, and indicate when practices diverge from expected care pathways. Education and provision of information to all involved participants were found to be facilitators, especially with the development of communities of practice, where a multidisciplinary team who shares the same goals work together to share new knowledge and lessons learned.^49^ Institutional resources such as creation of order sets and increasing front‐line staff are imperative to assure compliance and to make sure providers will have the ability to execute indicated orders, such as early mobilization for example. Moreover, assuring visibility of the protocol as well as providing constant updates back to staff were found to be important facilitators. Finally, setting up patient's expectations upfront and outlining to their family members what to expect after surgery were also found to drive not only adherence, but satisfaction rates as well. Consistency of information provided to patients may ultimately give them confidence to take care of themselves at home and make them feel better prepared for discharge from the hospital.

### 6.2 Implementation strategies

Given the complexity and high numbers of variables, ERAS guidelines require a designed execution strategy for successful implementation. Multiple guidelines have been published, but two framework strategies became very popular: breakthrough and knowledge‐to‐action (KTA) implementation strategies.^50^, ^51^ The breakthrough strategy entails having an external agent who would make site visits, the creation of multidisciplinary groups with scheduled meetings to discuss all innovations and then multiple learning sessions. This group works continuously using an act‐plan‐study‐do framework, supervised by external agents who support the whole process for about a year.^50^

Similarly, the KTA process is a collaborative method that involves both the creation and application of knowledge. Briefly, it involves identifying potential problems, adapting existing knowledge to local context, assessing barriers to knowledge use, and selecting tailored interventions with subsequent monitoring and evaluation of outcomes.^51^

Finally, since 2010 the ERAS society has stablished itself as a leader in the field and has issued multiple reviews and updates to facilitate the implementation of programs, including a guideline for perioperative care after RC for bladder cancer.^3^ Ideally, this should serve more so as a starting point and summary of evidence so that modifications can be made in order to adapt processes to local practices.

### 6.3 Evaluation of ERAS success

Implementation and maintenance of ERAS protocols depend on continued data collection, assessment of performance, and provision of feedback.^52^ With regards to data collection, the European ERAS Society and the American College of Surgeons National Quality Improvement Program (NSQIP) databases can both be used for monitoring and subsequent assessment of compliance with protocols. A systematic review of over a 100 randomized trials concluded compliance increases if institutions collect data both before and after implementation. Sharing data with all participants on a regular basis, establishes short‐term goals and provides feedback at rapid intervals so that changes can be made and reevaluated for usefulness, and if they have an ERAS team with enthusiastic coordinators and champions.^52^

After an initial period of rapid increase in compliance, most authors have shown a decrease in adherence in the following years after implementation.^53^ Interestingly, this well‐described decrease in conformity does not seem to be related to worse clinical outcomes, functional recovery or complication rates.^53^ Similarly, the impact on LOS despite a decrease in compliance with the protocol in the following 3‐5 years after implementation appears to be minimal.^50^ Since some providers demonstrate concerns regarding ERAS sustainability, it is very important to highlight that these observed trends in decreasing compliance over the years did not seem to negatively influence short‐term treatment outcomes.^50^

### 6.4 Continuous quality improvement with ERAS

As described above, a concern after implementation of ERAS protocols is sustaining it on day‐to‐day practice. Maintaining an implemented ERAS protocol and its benefits in the setting of a quality improvement collaborative (QIC) demands several planned activities and focused interventions for programmatic maintenance and longevity. A recent pooled analysis of post‐implementation data at multiple hospitals from the Netherlands identified potential strategies that could aid at sustaining ERAS outcomes.^54^ Even though there were large variations within the group of hospitals included in the analysis, the data showed that most were still maintaining LOS below the national average, only slightly increased compared to 3‐5 years earlier.^54^ In general, strategies should target both professionals and the organization. Methodologies that were found to impact the staff were continued internal audit and feedback on outcomes, small‐scale educational boosters, and constant reminders. On the other hand, the approaches that seemed to be helpful in sustaining benefits at an organizational level were the change in multiple care processes, delegation of responsibility and having multiple coordinators at different levels of care.^54^

Despite growing evidence of ERAS success, examining the correlation between individual interventions and adherence rates is very important, as part of quality improvement measures.^2^ One recent study proposed the creation of an importance‐performance matrix in order to prioritize areas for improvement.^55^ Quality evidence based protocols are key components of the ERAS. Performance is characterized by the adherence rate (number of patients that received an intervention/patient for whom the intervention was indicated). Remarkably, by combining these data on importance and performance, the authors were able to identify potential areas for improvement and that adherence does improve outcomes.^2^, ^55^

Another important aspect of ERAS is that constant change in practice eventually affects outcomes, like transformation in surgical techniques, development of newer non‐opioid strategies to treat post‐operative pain, etc. Therefore, compliance measures must change in order to account for changes brought in by the continuous evaluation of evidence and guideline modifications.

## 7 LIMITATIONS

While ERAS principles can be applied to almost any urologic surgeries, there are currently no high or moderate level of evidence for other very common surgeries for a comprehensive ERAS pathway.

## 8 CONCLUSION

ERAS requires multidisciplinary and multimodal approaches to surgical recovery. Although much of the focus has been in patients undergoing RC, the principles are widely applicable to almost all major urologic oncology surgeries. It is unknown which components have the greatest influence on hastening recovery and they may very based on local and organizational cultures. Implementation is possible in almost every setting, although diligence is required to continue to maintain and improve an ERAS program.
