# Supplementary material for: Enhanced recovery after surgery review and urology applications in 2020
Source: BJUI Compass. 2020 Mar 17;1(1):5–14. doi: 10.1002/bco2.9 (PMC8988792; doi:10.1002/bco2.9)
Supplement: Supplementary file 1 [file BCO2-1-5-s001.docx]

Key words used in the pubmed search

‘cystectomy’, ‘radical prostatectomy’, ‘nephrectomy’, ‘counseling’, ‘stoma education’, ‘optimization of medical condition’, ‘cessation of smoking and alcohol’, ‘prehabilitation’, ‘exercise’, ‘mechanical bowel preparation’, ‘preoperative fasting’, ‘carbohydrate loading’, ‘alvimopan’, venous thromboembolism prophylaxis’, ‘antibiotic prophylaxis’, ‘skin preparation’, ‘anesthetic protocols’, ‘surgical approach’, ‘perioperative fluid management’, ‘ nasogastric tube placement’, ‘prevention of intraoperative hypothermia’, ‘urinary catheter’, ‘pelvic drainage’, ‘postoperative nausea and vomiting’, ‘early mobilization’, ‘ileus prophylaxis’, ‘postoperative analgesia’, ‘early feeding’, ‘discharge’, ‘adherence’, ‘compliance’, ‘quality improvement’, ‘implementation’, ‘barriers’, and ‘ sustainability’
