# Supplementary material for: Enhanced recovery after surgery review and urology applications in 2020
Source: BJUI Compass. 2020 Mar 17;1(1):5–14. doi: 10.1002/bco2.9 (PMC8988792; doi:10.1002/bco2.9)
Supplement: Supplementary file 2 [file BCO2-1-5-s002.docx]

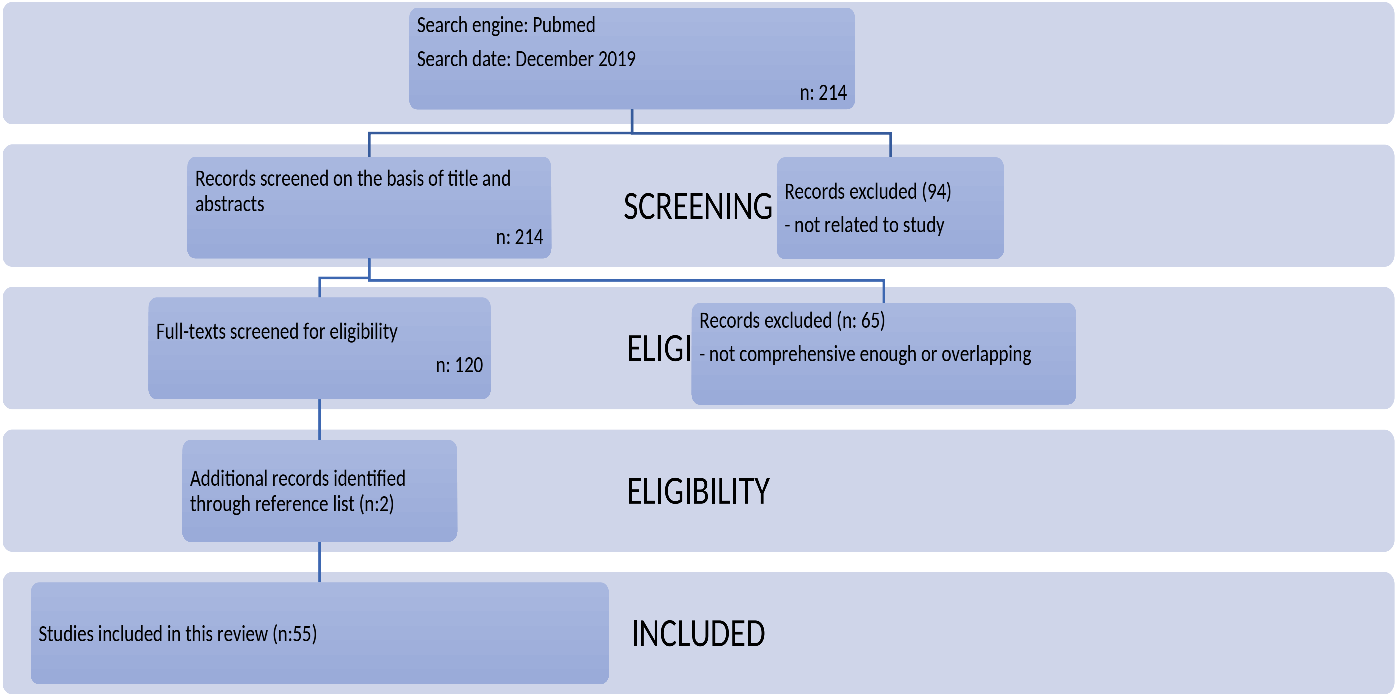
Search engine: Pubmed
Search date: December 2019
n: 214
Records screened on the basis of title and abstracts
n: 214
SCREENING
Records excluded (94)
- not related to study
Full-texts screened for eligibility
n: 120
ELIGI
Records excluded (n: 65)
- not comprehensive enough or overlapping
Additional records identified through reference list (n:2)
ELIGIBILITY
Studies included in this review (n:55)
INCLUDED
